# Matches

**Published:** 1860-12

**Authors:** 


### MATCHES.

THE fumes arising from Lucifer matches, says Dr. Hall's new book on SLEEP and the ventilation of chambers, are so destructive to the girls employed in the factories, that some of the European governments have taken measures to suppress them. Horrible ulcers form in the flesh, and fasten on the jaw-bone, eating it away by slow degrees. A single box of common matches will scent a room for several days; and children have been poisoned by eating them, as they have a sweetish taste. It is, therefore, a matter of public gratulation that a patent has been obtained for making strela matches—a Russian term for "lightning." They are without sulphur and without smell; are beautifully varnished, and are warranted to stand both damp and hot climates. A box of sulphur-matches contains eighty, and retails at one cent; a box of strela matches contains one hundred and fifty, at two cents. By the ten-gross case, they are wholesaled at one dollar and sixty cents per gross of one hundred and forty-four boxes. They light instantly and easily.

